# Identification of Immune-Related Breast Cancer Chemotherapy Resistance Genes *via* Bioinformatics Approaches

**DOI:** 10.3389/fonc.2022.772723

**Published:** 2022-03-21

**Authors:** Yabing Du, Yikai Han, Xin Wang, Huanrong Wang, Yanhong Qu, Kaiyuan Guo, Wang Ma, Lijun Fu

**Affiliations:** ^1^ Department of Oncology, The First Affiliated Hospital of Zhengzhou University, Zhengzhou, China; ^2^ Department of Radiotherapy, The First Affiliated Hospital of Zhengzhou University, Zhengzhou, China; ^3^ Oncology Department of Laiyang People’s Hospital, Laiyang, China; ^4^ Department of Thyroid Surgery, The First Affiliated Hospital of Zhengzhou University, Zhengzhou, China

**Keywords:** breast cancer, chemotherapy resistance, immune microenvironment, PRC1, GGTLC1, IRS1

## Abstract

Chemotherapy resistance in breast cancer is an important factor affecting the prognosis of breast cancer patients. We computationally analyzed the differences in gene expression before and after chemotherapy in breast cancer patients, drug-sensitive groups, and drug-resistant groups. Through functional enrichment analysis, immune microenvironment analysis, and other computational analysis methods, we identified PRC1, GGTLC1, and IRS1 as genes that may mediate breast cancer chemoresistance through the immune pathway. After validation of certain other clinical datasets and *in vitro* cellular assays, we found that the above three genes influenced drug resistance in breast cancer patients and were closely related to the tumor immune microenvironment. Our finding that chemoresistance in breast cancer could be influenced by the mediation of tumor immunity expanded our knowledge of how to address this problem and could guide future research involving chemoresistance.

## Introduction

Breast cancer surpassed lung cancer as the most common type of malignancy worldwide in 2020 (1), while it remains the leading cause of cancer death among women. There have been many advances in treating it in the past decades, such as surgery, radiation therapy, chemotherapy, endocrine therapy, and targeted therapy (2). Chemotherapy is important and effective in the treatment of treatment. Consequent chemotherapy resistance is an important obstacle in the successful treatment of breast cancer, especially in metastatic breast cancer where the vast majority of treatment failures are due to chemotherapy resistance (3). Therefore, chemotherapy resistance occurs in a significant fraction of patients (4) leading to disease progression and ultimately death. It has been thought that chemotherapy resistance is the result of intrinsic cell genetic changes, including upregulation of drug efflux pumps, activation of detoxifying enzymes, or apoptotic defects (5). However, over the past 10 years, increasing evidence has shown that chemotherapy resistance is also related to tumor microenvironment (6).

Tumor immune microenvironment (TIME) refers to the biological interactions between tumor, stroma, and immune cells, and a deep understanding of the TIME contributes to the success of clinical treatment (7). As important parts of the TIME, disrupted balance of growth factors, chemokines, cytokines, immune cells, and stromal cells have been recognized as some of the important mechanisms of chemotherapy resistance (6).

Chemotherapy resistance mediated by the TIME is a complex process that requires the involvement of not only immune cells, but also protein molecules and cytokines on the tumor surface. Even differences in the expression levels of genes in tumor cells and immune cells can result in differential responsiveness to chemotherapeutic agents. These genes were previously thought to be intrinsic to chemoresistance, and a growing number of studies have suggested that they may mediate chemotherapy resistance by affecting the immune microenvironment. For example, overexpression of CD137 in osteosarcoma is associated with chemosensitivity (8), possibly because CD137 can induce immune clearance of the tumor by the microenvironment. In addition, overexpression of PDGFD (9, 10), a provascular growth factor in ovarian and colon cancers, predicts chemoresistance, and exosomes secreted by Mesenchymal Stem Cells(MSCs) can affect S100 Calcium Binding Protein A6(S100A6)expression to mediate chemotherapy resistance in breast cancer (11).

In addition, the TIME affects the response of tumor cells to chemotherapeutic agents in several ways: 1) Various immune factors can cause cellular autophagy, which can affect the anti-tumor response of natural killer cells (12). 2) Multiple factors can lead to changes in the ratio of Treg cells, CD8+ T cells, cytotoxic T lymphocytes (CTL cells), myeloid-derived suppressor cells (MDSCs), and macrophages, as well as changes in the expression of cell surface protein molecules such as programmed death ligand 1 (PD-L1), CTLA-4, and CD47, ultimately leading to the development of an immunosuppressive microenvironment (13–15). 3) The immune microenvironment leads to activation of intracellular signaling pathways that can generate chemoresistance, for instance, activation of the AKT/ERK signaling pathways can induce expression of anti-apoptotic genes (16). 4) Two immune microenvironment cytokines, IL-6 and tissue inhibitor of metalloproteinase 1 (Timp-1), can protect tumor cells from cell death induced by genotoxic chemotherapy. IL-6 and Timp-1 can generate chemoresistance by affecting the immune microenvironment, a situation that has been demonstrated in lymphoma and hepatocellular carcinoma (6).

Researchers recently constructed a signature associated with FOLFIRI resistant and Microenvironment (SFM) of colon cancer chemotherapy-associated features by computational statistical analysis of data from multiple pools of colon cancer patients receiving chemotherapy (17). The composition of the immune microenvironment varied in the different SFM classifications, for example, SFM-D exhibited enrichment with activated naive CD4 T and B cells, plasma cells, CD8 T cells, and Tregs. SFM-E showed increases of follicular helper T cells, M0/1 macrophages, and neutrophils. Ultimately, these classifications correlated closely with clinical survival in colon cancer patients receiving chemotherapy, demonstrating that the immune microenvironment may also be an important cause of chemoresistance in colon cancer patients.

There are few studies on the influence of the immune microenvironment on chemoresistance in breast cancer patients. Most of them have focused on establishing the corresponding genetic profiles without any in-depth studies on why these genetic differences caused the varied responses of breast cancer cells to chemotherapeutic drugs and with little research on the analysis of immune mechanisms. In the present study, we used computational analysis and machine learning methods to find genes that may contribute to chemotherapy resistance by the immune pathway, and we provided our perspective on immune-mediated chemotherapy resistance in breast cancer.

## Materials and Methods

The flow chart showing the overall research design and methods used for this study is shown in **Supplementary Figure 1**.

### Data Download and Preliminary Data

The GSE28694 (Miller and Payne grades 4 and 5) and GSE28826 (Miller and Payne grades 1 and 2) chip data were downloaded from the GEO database containing a total of 41 samples. Among them, 28 samples were grades 1 and 2 of the Miller-Payne classification system, and 13 samples were grades 4 and 5. Miller-Payne grades 1 and 2 (Grades12) were defined as drug resistance groups, and Miller-Payne grades 4 and 5 (Grades45) were defined as drug-sensitive groups. These data were used as training sets.

We standardized the chip data according to the Robust Multichip averaging method (18). The expression value of each gene was calculated based on the correspondence between the probe and the gene. For the case where a gene corresponded to multiple probes, we chose the average of these probes as the expression value of the gene.

### Screening Differentially Expressed Genes

The gene expression data was analyzed for differential expression using the R package limma, and the genes that expressed differences in the samples were screened according to the difference multiple (|logFC| > 0.585) and the significance level (P value < 0.05). The sample grouping was based on the following:

a) Compare samples before chemotherapy in Grades12 and Grades45
b) Compare the samples after chemotherapy in Grades12 and Grades45
c) Compare samples before and after chemotherapy in Grades12
d) Compare samples before and after chemotherapy in Grades45

### Functional Enrichment Analysis

ClusterProfiler was used to perform enrichment analysis and visualization of GO function and KEGG pathway.

### Establishing a Protein Interaction Network of Differential Genes

According to STRING 11.0, we established a protein interaction network of differential genes and performed network enrichment analysis (Cytoscape string application). We then assessed the aggregation level of the differential protein interaction network by constructing a zero distribution of the network aggregation level. Specifically, the same number of genes as the differential genes were randomly selected from the protein-interaction network and the number of connected edges of these genes was calculated. We randomly generated (x1000) a distribution of the number of edges in a random partition to determine whether the edges in the differential protein interaction network were significant. High-functional enrichment analysis uses similar and random comparison methods for identification involving GO, KEGG Pathways, and the Reactome Pathways database. Among them, GO functional enrichment only focuses on biological processes (BP).

### Optimizing the Identification of Potential Related Genes

Using drug resistance-related genes, cancer stem cell markers, and the ABC transport family genes as seed samples, we used the random walk method to evaluate the association of differential genes with the above-mentioned gene set for screening. A random network was generated repeatedly through the interference dynamics network, and the calculation was repeated (x1000) to generate a random distribution of gene scores, thereby calculating the significance P value. Genes with P < 0.01 were extracted as genes with significant association.

The recognized related pathways were retrieved from the KEGG database, including four correct related pathways, namely platinum resistance (hsa01524), antifolate resistance (hsa01523), endocrine resistance (hsa01522), and the ABC transporter (hsa02010) chemical. If a gene was significant in more than two random walk results, we extracted the gene to replace the related gene set, and finally we extracted GGTLC1, IRS1, and PRC1 by calculation.

### Tumour Immune Microenvironment

#### Immune Signature

According to the standardized expression profile data, the geometric average method was used to calculate the TIME characteristics of the sample, including “adhesion molecule,” “chemokine,” “cell decomposition activity,” “IFN-γ signature,” “Immune costimulator,” “Immunosuppressant,” and “MHC Class I.”

#### Cancer Immune Cycle

Based on the standardized expression profile data, the ssGESA method was used to calculate the cancer immune loop, including “Step 1: Cancer cell antigen release,” “Step 2: Cancer antigen presentation,” “Step 3: Activation and activation,” “Step 4: Immune cell trafficking,” “To the tumor,” “Step 5: Immune cells infiltrate the tumor,” “Step 6: T cells recognize cancer cells,” and “Step 7: Kill cancer cells.” The cancer immune cycle score calculated by ssGSEA was not comparable among samples, so we normalized the immune cycle score of each sample based on random background. Specifically, the random disturbance expression matrix used the same calculation method to calculate the random cancer immune cycle score. This was repeated (x100) to generate a random cancer immune cycle score distribution. We then integrated the real sample and random data and used the zscore method to calculate the score of the real sample relative to random.

#### Immune Cell Infiltration

According to the standardized expression profile data, the CIBERSORT method was used to analyze the proportion of 22 immune cell infiltrations in tumor samples, including B cell naive, B cell memory, plasma cells, T cell CD8, T cell CD4 naive, T cell CD4 memory quiescent, T cell CD4 memory activation, T cell follicular assist, T cell regulation (Treg), T cell gamma δ, resting NK cells, activated NK cells, monocytes, macrophages M0, macrophages M1, macrophages M2, Dendritic cells are stationary, dendritic cells are activated, mast cells are resting, mast cells are activated, eosinophils, and neutrophils.

Using the consistent clustering method (R package Consensus ClusterPlus), the patients were divided into multiple immune subtypes. This was repeated (x1000) in the consistent clustering method. Considering the two indicators of CDF and Delta regions at the same time, we determined the optimal clustering diversity (cancer immune cycle, category 3; immune cell infiltration, category 4).

Wilcox grades and tests were used for comparative analysis between Grade 12 and Grade 45 pre-chemotherapy samples.

### Creating a Correct Machine Learning Model for Patients

For each patient, we constructed 15-dimensional features. We calculated the transformation path score of the sample using the geometric mean method and identified the relevant gene score and the immune signature score. A random forest classifier consisting of 1000 trees was formed using random forests. Each tree was reconstructed by randomly selecting the same number of negative pairs as the positive set, using the R package randomForest.

### Verifying the Characteristics of the Three Genes *In Vitro*

#### Cell Lines and Cell Culture

The human breast cancer cell line MDA-MB-468 was used for all experiments, which was obtained from the Cell Bank of the Chinese Academy of Sciences (Shanghai, China). The cells were cultured in RPMI 1640 (Bioss) supplemented with 10% FBS (Biological Industries), 1% penicillin, and 1% streptomycin (Biosharp) at 37°C in a 5% CO_2_-humified atmosphere.

#### Cell Transfection

For siRNA-mediated knockdown, cells were seeded at 1.5 × 10^5^ per well in 6-well plates 24 h before use. According to the manufacturer’s recommendations, when the cells reached 70% to 90% confluence, we transfected the cells with Lipofectamine 3000 (Invitrogen, Waltham, MA, USA) in a serum-free medium for 6 h. After that, the siRNA was removed, and the cells were cultured for 48 h in a regular medium. The following siRNAs were obtained from Shanghai GenePharma Co. Ltd: IRS1 (IRS1-Homo-2025), PRC1 (PRC-Homo-1047), and GGTLC1 (GGTLC1-Homo-504). For control knockdown, Negative Control (GenePharma, Shanghai, China) was used. All the above RNA oligo sequences are presented in the Supplementary material.

#### Quantitative Real-Time Polymerase Chain Reaction

The TRIzol reagent (Invitrogen, China) was used for extraction of total RNA from the cells according to the manufacturer’s instructions. The First-strand cDNA was synthesized from 1 μg of total RNA using HiScript III RT SuperMix for qPCR (+gDNA wiper) (Vazyme, China). qRT-PCR was performed using ChamQ Universal SYBR qPCR Master Mix (Vazyme, China) with an Applied Biosystems (USA) instrument. Sequences of the primers used for qRT-PCR are listed in the Supplementary material. GAPDH was used for normalizing the ΔΔCt values of the studied genes.

#### Cell Counting Kit-8 Assay

MDA-MB-468 cells were seeded into 96-well plates at 8000 cells per well and incubated overnight. After 48 h of transient transfection according to the above method, the cells were treated with different concentrations of epirubicin (Macklin, China) for 48 h, or the cells were treated with different concentrations of paclitaxel (Macklin, China) for 36 h and then were assessed for viability using a CCK-8 reagent (APExBIO, USA) as per the manufacturer’s manual. Briefly, 10 μL of the CCK-8 reagent was added to each well of the 96-well plates, which were incubated for 2 h at 37°C. The optical density (OD) value at 450 nm was measured using a SpectraMax Absorbance Reader (Molecular Devices, CMax Plus, USA).

## Results

### Differential Gene Screening

We downloaded the GSE28694 (Miller & Payne grades 4 and 5, Grade45), GSE28826 (Miller & Payne grades 1 and 2, Grade12) chip data from the GEO database, which contained 61 samples. Among them, Grade12 contained 14 samples before and after chemotherapy; Grade45 contained 8 samples before and 5 samples after chemotherapy.

**Figure 1:**
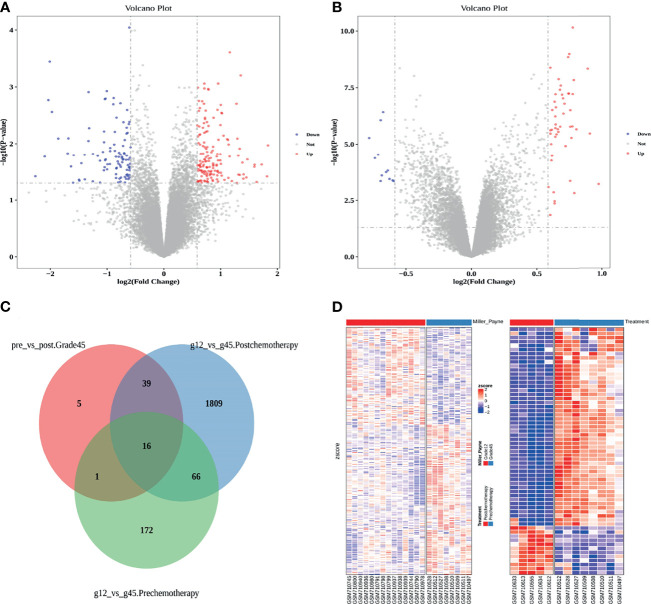
**(A)** Differentially expressed genes in Grade12 versus Grade45 before chemotherapy. **(B)** Grade45 differentially expressed genes before and after chemotherapy. **(C)** A Venn diagram of each group of samples comparing differentially expressed genes. **(D)** Comparison of Zscore for differential gene expression, Grade12 vs. Grade45 on the left and before and after treatment on the right.

By comparing Grade12 and Grade45 samples before chemotherapy, we identified 255 differentially expressed genes, including 155 upregulated genes and 100 downregulated genes (**Figures 1A, C, D**: **Table 1** and **Table S1**). Since Grade45 was the drug-sensitive group and Grade12 was the drug-resistant group, it is reasonable to believe that the differentially expressed genes identified in these two groups of samples before chemotherapy could represent the difference in their response to drugs; therefore, we defined these differentially expressed genes as potential drug-resistance genes (PDRGs). By comparing the samples before and after chemotherapy, we found 61 differentially expressed genes in Grade45, but no differentially expressed genes in the Grade12 samples (**Figures 1B–D**; **Table 1** and **Table S2**). These 61 genes could be considered as potential drug-induced genes (PDIGs).

**Table 1:** Statistics of the number of differentially expressed genes.

Pre-treatment vs. Post-treatment			
Group	Up	Down	Total
Grade 12	0	0	0
Grade 45	49	12	61
Grade 12 vs. Grade 45			
Group	Up	Down	Total
Pre-treatment	155	100	255
Post-treatment	948	982	1930

### Functional Enrichment Analysis

**Figure 2:**
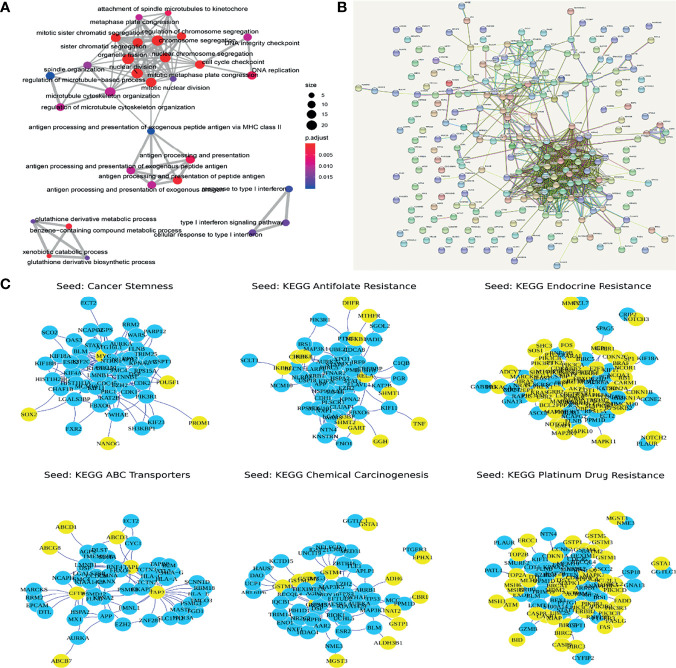
**(A)** Functional enrichment of genes with potential functions in drug response. **(B)** Protein interactive networks of differentially expressed genes. **(C)** Top 50 genes using random walk algorithm (yellow nodes are seed nodes).

Through GO enrichment analysis of the PDRGs, we identified 60 significantly related GO terms (FDR < 0.05, **Figure 2A**), including cell cycle, antigen presentation, and glutathione metabolism. It is worth noting that 33% (20/60) of the GO term was related to tumor immune response, and 18% (11/60) of the GO term was related to cell cycle. KEGG pathway enrichment analysis showed that drug response-related genes were significantly correlated with the drug metabolism-other enzymes pathway (FDR < 0.05, **Figure 2A**). In addition, the PDIGs intensively enriched cell cycle-related GO functions, as well as the p53 signaling pathway (**Figure 2A**). Both the PDRGs and PDIGs showed a strong enrichment of immune-related functions, and this result demonstrated the underlying mechanisms of breast cancer resistance to chemotherapy.

To optimize the identification of potential resistance-related genes, the random walk method was used to calculate the correlation between the dysregulated genes and the collection of known resistance-related genes. Genes that were significantly related in two or more sets were extracted as drug resistance-related gene sets (Potential Set, GGTLC1, IRS1, PRC1) (**Figures 2B, C**).

### Exploring Breast Cancer Chemotherapy Resistance From the Perspective of the Immune Microenvironment

**Figure 3:**
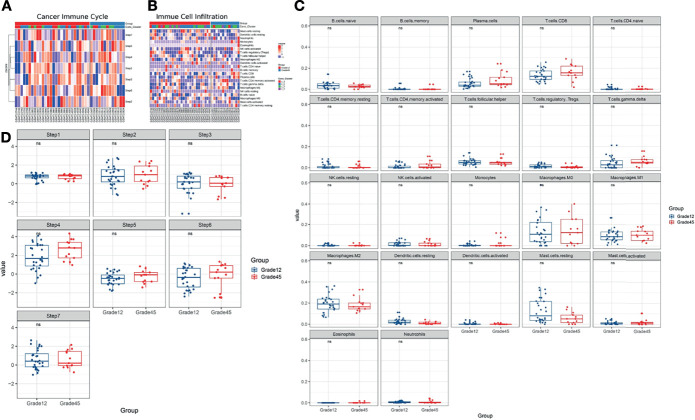
**(A, B)** Using Cancer Immune Cycle and Immune Cell Infiltration to evaluate the tumor immune microenvironment. **(C)** CIBERSORT method is used to analyze the proportion of 22 immune cells infiltration in tumor samples. **(D)** The ssGESA method is used to calculate the cancer immune circle. ns, no significance.

Based on the above results, we used the cancer immune cycle and immune cell infiltration to assess the TIME of the patients (**Figures 3A, B**) and the results showed that cancer immune cycle and immune cell infiltration did not show a significant difference between the two groups (**Figures 3C, D**).

Based on the collection of known immune-related genes, the differences in the TIME characteristics of Grade12 and Grade45 samples were further evaluated. It was found that the MHC Class I, cytolytic activity, and immuno-costimulators of the Grade12 samples were significantly lower than those in the Grade45 samples. Their reduction was related to the decrease of tumor antigen presentation and immune cell killing, which may have contributed to breast cancer chemotherapy resistance.

### Building a Predictive Model of Breast Cancer Chemotherapy Resistance

**Figure 4:**
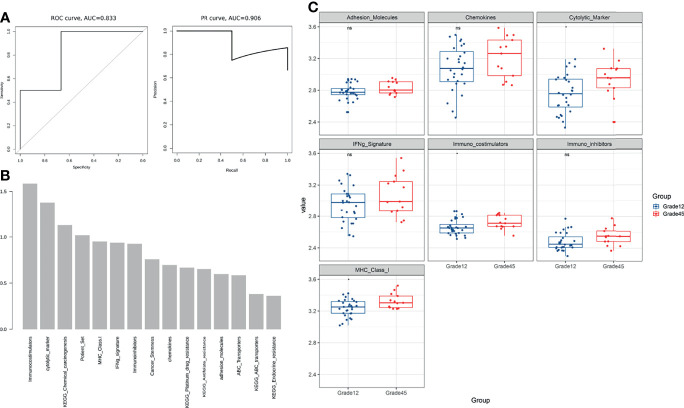
**(A)** The five-fold cross-validation results showed that the model could effectively identify patients with drug resistance. **(B)** The feature importance results show that three of the top 30 features are immune-related features **(C)** Comparison of immune characteristics between Grade 12 and Grade 45. ns, no significance.

For each patient, we calculated the resistance-related pathways, immune characteristics, ABC transporter, cancer stemness, and optimized resistance-related gene signature scores to construct a 15-dimensional feature matrix (**Table 2**). A random forest was used to build a predictive model of breast cancer chemotherapy resistance. The five-fold cross-validation results showed that the model could effectively identify patients with drug resistance (**Figure 4A**). The results of the feature importance indicated that three of the top 30 features were immune-related features (**Figure 4B**), highlighting the potential role of the immune microenvironment in breast cancer chemotherapy resistance.

**Table 2:** Features used in random forest.

Category	Name	Gene Number
Drug Resistance Pathway	KEGG_Platinum_drug_resistance	73
	KEGG_Antifolate_resistance	31
	KEGG_Endocrine_resistance	98
	KEGG_ABC_transporters	45
	KEGG_Chemical_carcinogenesis	82
Immune Signature	MHC Class-I	7
	Adhesion Molecules	6
	Chemokines	5
	Cytolytic Marker	4
	IFNg Signature	6
	Immuno-costimulators	17
	Immuno-inhibitors	12
Other	ABC_Transporters	4
	Cancer_Stemness	13
	Potential_Set	3

### External Data Verification of Candidate Biomarkers Related to Drug Resistance

Three candidate resistance-related genes were identified based on the discovery data set, including IRS1, PRC1, and GGTLC1.

IRS1 is an important signaling protein that participates in the regulation of important cancer-related pathways, such as the PI3K/AKT signaling pathway. Studies have shown that silencing IRS1 could enhance chemotherapy sensitivity in patients with breast and pancreatic cancer (19, 20). PRC1 is a microtubule-associated protein that plays an important role in cell mitosis and cell cycle regulation. In liver cancer, PRC1 has been found to be abnormally associated with chemotherapy resistance (21). GGTLC1 has not yet been studied to confirm its association with chemotherapy.

By searching the GEO database and extracting TCGA BRCA data, a total of five independent verification data sets were obtained (**Table 3**). For the TCGA BRCA data, only samples in which the treatment method was chemotherapy and had data on drug evaluation were extracted.

**Table 3:** Distribution about validation data sample.

Dataset	Sensitive	Resistant	Total
TCGA	166	21	187
GSE20271	26	152	178
GSE25055	57	249	306
GSE22093	28	69	97
GSE23988	20	41	61

**Figure 5:**
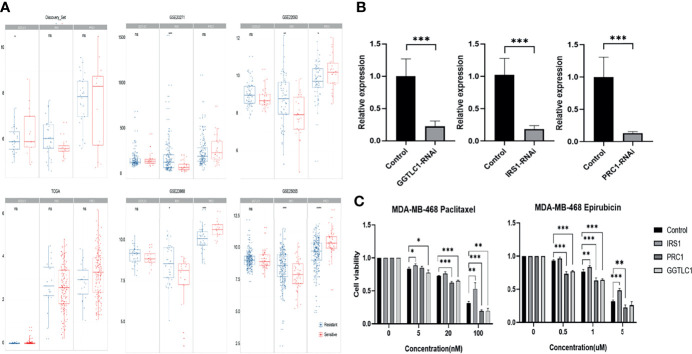
**(A)** Comparison of GGTLC1, PRC1, and IRS1 gene expressions in each data set. **(B)** Knockdown efficiency for GGTLC1, IRS1, and PRC1 were identified by qRT-PCR analysis. qRT-PCR, quantitative real-time polymerase chain reaction; *P < 0.05, **P < 0.01, ***P < 0.001. **(C)** Evaluate the cellular sensitivity to chemotherapeutics of decreased expression of IRS 1, PRC1, and TLC1 by the method of CCK-8. CCK-8: Cell Counting Kit-8, *P < 0.05, **P < 0.01, ***P < 0.001. ns, no significance.

In the four GEO independent verification sets, IRS1 was significantly increased in the drug resistance group, and PRC1 was significantly decreased in the drug resistance group (**Figure 5A**, Wilcoxon rank-sum test, P value < 0.05). Although there were no significant differences in the TCGA data, the trend of expression change was consistent.

Using the same data processing and calculation methods, the 15-dimensional features of the independent verification set were calculated and compared for analysis. The results showed that MHC Class-I (antigen presentation) was downregulated in four independent verification sets, while the cytolytic marker (T cell killing) was downregulated in three independent verification sets (**Table 4**, **Figure 4C** and **Figures S2** **–** **S6**).

**Table 4:** Comparing the resistant group and the sensitive group, the immune-related characteristics are downregulated.

Features	Discovery Set	TCGA	GSE20271	GSE22093	GSE25055
MHC_Class-I	Down	Down	Down	Down	Down
Adhesion Molecules			Down		
Chemokines			Down	Down	Down
Cytolytic Marker	Down		Down	Down	
IFNg Signature		Down	Down		Down
Immuno- costimulators	Down				
Immuno- inhibitors		Down			Down

We then used these data to verify the effectiveness of the model. Unfortunately, the size of the training sample was limited, and the performance of the current model was poor (**Table 5**). In addition, modeling based on large sample data sets (TCGA, GSE20271, and GSE25055) did not improve the model’s effectiveness, which may be due to the extreme imbalance between negative and positive samples (data not shown). TCGA data is based on RNA-seq, while the data from GEO sources are based on HGU133 chip data, due to platform differences, and cannot be integrated.

**Table 5:** Comparing the resistant group and the sensitive group, the immune-related characteristics are downregulated.

Dataset	AUC_ROC	AUC_PR
TCGA	0.6757028	0.2521503
GSE20271	0.6690283	0.9203683
GSE25055	0.6144986	0.8562434
GSE22093	0.5523349	0.7536336
GSE23988	0.5792683	0.6485242

Finally, based on the results of five validation data sets and the support of existing literature, we hypothesized that IRS1 and PRC1 may play an important role in breast cancer resistance. In addition, all the data showed the important role of the immune microenvironment in drug resistance, and this phenomenon has also been observed in cancers such as lung cancer and gastric cancer (22, 23). Moreover, recent clinical studies have shown that combined immunotherapy and chemotherapy could greatly improve the overall survival of patients and support the potential role of immune microenvironment and chemotherapy (24, 25).

### *In Vitro* Cell Experiments Verify That the Three Genes Are Associated With Breast Cancer Drug Resistance

SiRNA can significantly knockdown the expression of the corresponding gene (**Figure 5B**). Decreased expression of IRS1 increased the resistance of MDA-MB-468 cells to epirubicin and paclitaxel. In contrast, the decreased expression of GGTLC1 and PRC1 increased the sensitivity of MDA-MB-468 cells to epirubicin and paclitaxel (**Figure 5C**). The cell experiment results of GGTLC1 and PRC1 were consistent with the calculation analysis, while the cell experiment results of IRS1 were different from the calculation analysis.

## Discussion

We identified 255 differentially expressed genes by comparing breast cancer chemotherapy-resistant samples with breast cancer sensitive samples and identified 61 differentially expressed genes by comparing breast cancer sensitive samples before and after chemotherapy. Through functional enrichment analysis, we found most of the differentially expressed genes were related to tumor immune response. Through the random walk method, we finally identified that GGTLC1, PRC1, and IRS1 may have produced breast cancer drug-resistant phenotypes through immune-mediated pathways. Subsequently, the above results were also verified through external data sets. We hypothesized that immune pathways could indeed affect the chemotherapy resistance of breast cancer.

GGTLC1 is a member of the glutamyl transferase family, encoding the light chain part of GGT, which is the catalytically active part of the GGT1 protein. It was called GGTL6, GGTLA4 before being named GGTLC1 in 2008 (26). Ovarian cancers overexpressing GGT1 showed greater resistance to chemotherapy, especially cisplatin (27) and 5-fluorouracil (28). Inhibiting the function of GGT1 can significantly inhibit the metastasis of renal clear cell carcinoma and improve the sensitivity to chemotherapy (29). Kawakami et al. (30) found that GGT1 could also be used as a biomarker to distinguish prostate cancer from benign prostate tumors (30). The prognosis of breast cancer patients with negative GGT1 expression was better than that of breast cancer patients with positive GGT1 expression. GGT1 may promote drug efflux, affect glutathione metabolism and cellular redox status, and regulate the cell cycle to produce chemotherapy resistance. Additionally, in lung cancer models, GGT1 promoted the metabolism of LTC4 to LTD4, which could promote lung inflammation and tumorigenesis (31).

In hepatocellular carcinoma, GGT1 expression has been positively correlated with the level of infiltration of CD4+ T cells, macrophages, and dendritic cells (32). In addition, GGT1 was linked to the T cell receptor signaling pathway. Abnormal expression of GGT family proteins, including GGT1, could cause increased oxidative stress within tumor cells, which affects the TIME and influences the response to chemotherapeutic agents. Recently, Li (33) found that increased expression of GGT1 in triple negative breast cancer caused cisplatin resistance by affecting the hepatic leukemia factor (HLF), a process that may be closely linked to IL-6 levels in the immune microenvironment (33). In this complex process, GGT1 interacted with components of the immune microenvironment to influence TNBC proliferation, invasion, and platinum resistance.

Protein regulator of cytokinesis 1 (PRC1) encodes a protein involved in cytokinesis. This protein is expressed at high levels during the S and G2/M phases of mitosis, but its level drops sharply when the cell enters the G1 phase. PRC1 has been shown to be a substrate for several cyclin-dependent kinases (CDK) (34, 35). Wang et al. (21) found that the high expression of PRC1 in hepatocellular carcinoma mediated 5-fluorouracil resistance by affecting the cell cycle (21). PRC1 plays an important role in the carcinogenesis of bladder cancer. The study results of Kanehira et al. (36) showed that knockdown of PRC1 expression with specific small interfering RNAs caused a significant increase of multinuclear cells and subsequent cell death of bladder cancer cells (36). PRC1 gene knockdown can reduce the proliferation, metastasis, and multidrug resistance of ovarian cancer cells (37). In previous studies, the overexpression of PRC1 mediated the early recurrence of hepatocellular carcinoma through the Wnt/β-catenin signaling pathway and increased the resistance of HCC to paclitaxel (38, 39). Additionally, in gastric and lung cancers, the high expression of PRC1 has often been associated with early lymph node metastasis and poor prognosis (40, 41). Previous bioinformatics analyses have revealed that PRC1 was associated with immune invasion of hepatocellular carcinoma (42).

Insulin receptor substrate 1 (IRS-1) is the first member of the insulin receptor substrate (IRS) protein family to be identified. It is located in the cytoplasm and can integrate a variety of cell biological functions. It is now clearly known that IRS1 is the main substrate of insulin-like grow factor 1 receptor (IGF-1R) (43, 44). IRS1 itself has no kinase activity; however, after stimulation by upstream signals, multiple tyrosine kinase sites are phosphorylated, which in turn affect multiple downstream signaling pathways. The two most studied are the PI3K/Akt/mTOR and the MAPK pathways, which can ultimately affect the invasion and metastasis of tumors (45).

Our computational analysis found that increased expression of IRS1 may lead to breast cancer resistance, which is inconsistent with the results of previous cell experiments. In fact, the results of research on the effect of IRS1 on drug resistance were not consistent, and most studies showed that the decline in IRS1 expression promoted the development of breast cancer drug resistance. In addition, the upregulation of IRS1 expression promoted the activation and proliferation of CD4+ T cells and the secretion of IFN-γ (46). The expression of IRS1 in breast cancer cells varies with tumor invasiveness. Differential expression may affect the prognosis of breast cancer patients. Further, IRS1 sensitized BC cells to specific chemotherapeutic drugs, and decreased expression of IRS1 enhanced the resistance of BC cells to paclitaxel, etoposide, and vincristine, but did not change the sensitivity of BC cells to doxorubicin, camptothecin, and daunoblastina (47). This may be one of the reasons why the results of our cell experiments were inconsistent with the results of the computational analysis. Another reason is the computational analysis of data from clinical samples of patients was affected by many factors, not just the expression levels of the genes, which also reflects the limitations of bioinformatics analysis.

Synthetically, both GGTLC1 and IRS1 can affect the levels of cellular components and cytokines in the immune microenvironment. GGTLC1 affected IL-6 levels and influenced changes in the proportion of various cells, including CD4+ T cells and macrophages in the TIME. In addition, GGTLC1 was also closely related to oxidative stress, ROS production, and tumor cell proliferation. Abnormal expression of PRC1, a protein molecule that plays a key role in cell division, has been linked to the proliferation of tumor cells and even normal cells, and was also thought to affect the process of oxidative stress and participate in the Wnt/β-catenin signaling pathway. IRS1 is closely related to the immune microenvironment and can affect CD4+ T cells and IFN-γ levels, and IRS1 was associated with resistance to multiple chemotherapeutic agents in breast cancer cells. We therefore hypothesized that the roles of these three may be interlinked, with GGTLC1 and PRC1 sharing a common role for cellular oxidative stress and cell proliferation and division. GGTLC1 and IRS1 could jointly influence changes in components of the immune microenvironment, with all three associated with a survival benefit for patients. One of the possible reasons for this is through the influence of chemotherapy resistance, an aspect that requires more in-depth research.

## Conclusion

We discovered three genes: PRC1, GGTLC1, and IRS1 that may mediate breast cancer chemotherapy resistance through immune pathways and found that immune regulation disorders may be some of the key factors in the survival of breast cancer patients.

## Data Availability Statement

The original contributions presented in the study are included in the article/**Supplementary Material**. Further inquiries can be directed to the corresponding authors.

## Author Contributions

YD and YH designed and conducted the experiments, analyzed and interpreted the data, and wrote the manuscript. XW and YQ performed the biological analysis of the data. HW performed some of the cell experiments and advised on the manuscript. LF guided the experiments and revised the manuscript. WM conceived the project, supervised the experimental design and data interpretation and wrote the manuscript. All authors contributed to the article and approved the submitted version.

## Funding

Natural Science Foundation of Henan Province (Youth Science Foundation Project) 202300410402. Young Elite Scientists Foundation Project of Henan Province (2021HYTP058).

## Conflict of Interest

The authors declare that the research was conducted in the absence of any commercial or financial relationships that could be construed as a potential conflict of interest.

## Publisher’s Note

All claims expressed in this article are solely those of the authors and do not necessarily represent those of their affiliated organizations, or those of the publisher, the editors and the reviewers. Any product that may be evaluated in this article, or claim that may be made by its manufacturer, is not guaranteed or endorsed by the publisher.

## Supplementary Material

Supplementary Figure 1 The workflow of our study.

Supplementary Figure 2-6 Comparison of differences in immune characteristics between the drug-resistant group and drug-sensitive group.
