# Supplementary figures and images for: Identification of Immune-Related Breast Cancer Chemotherapy Resistance Genes via Bioinformatics Approaches
Source: Front Oncol. 2022 Mar 21;12:772723. doi: 10.3389/fonc.2022.772723 (PMC8978268; doi:10.3389/fonc.2022.772723)

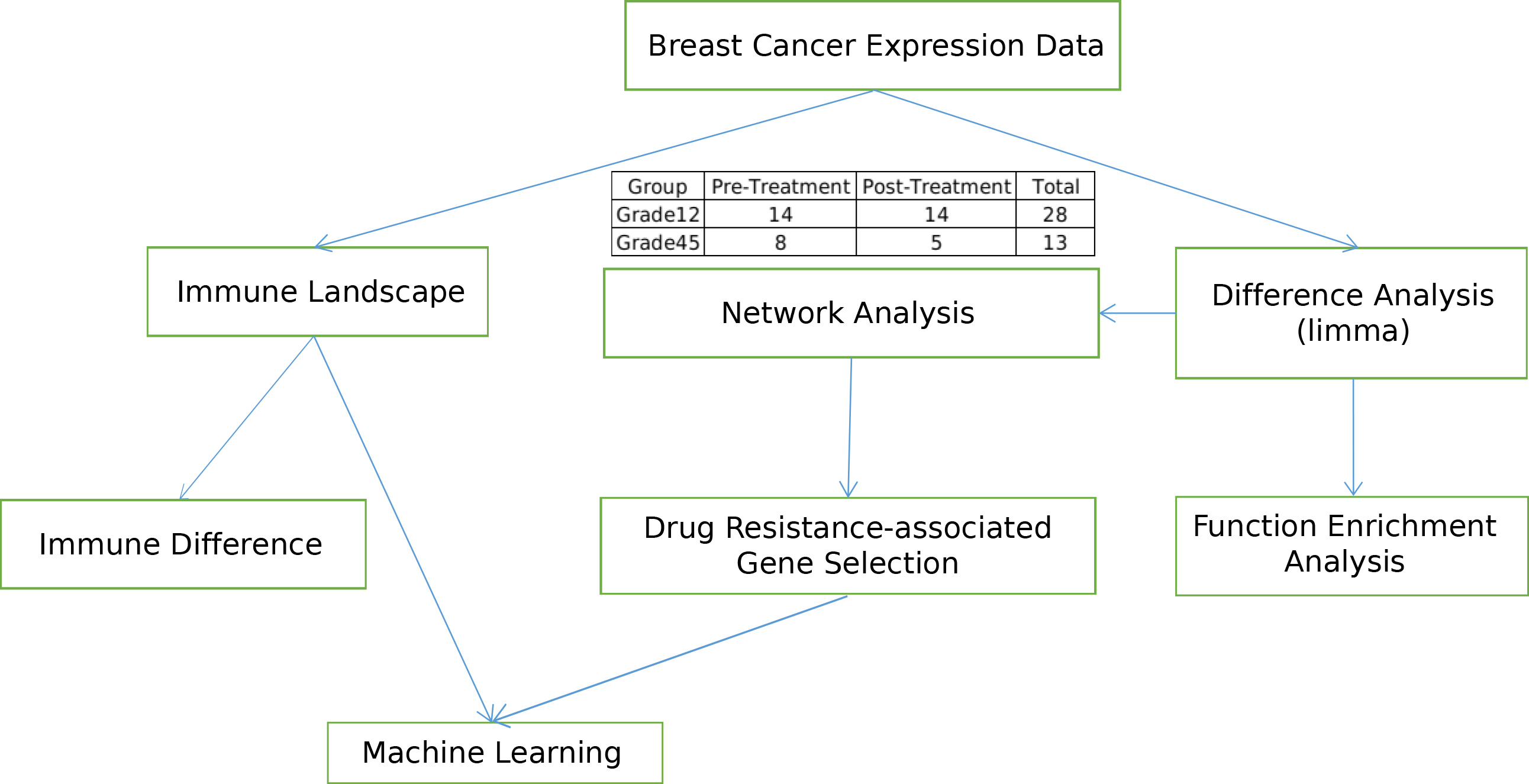

Supplement: Supplementary Figure 1 — The workflow of our study. [file Image_1.jpeg]

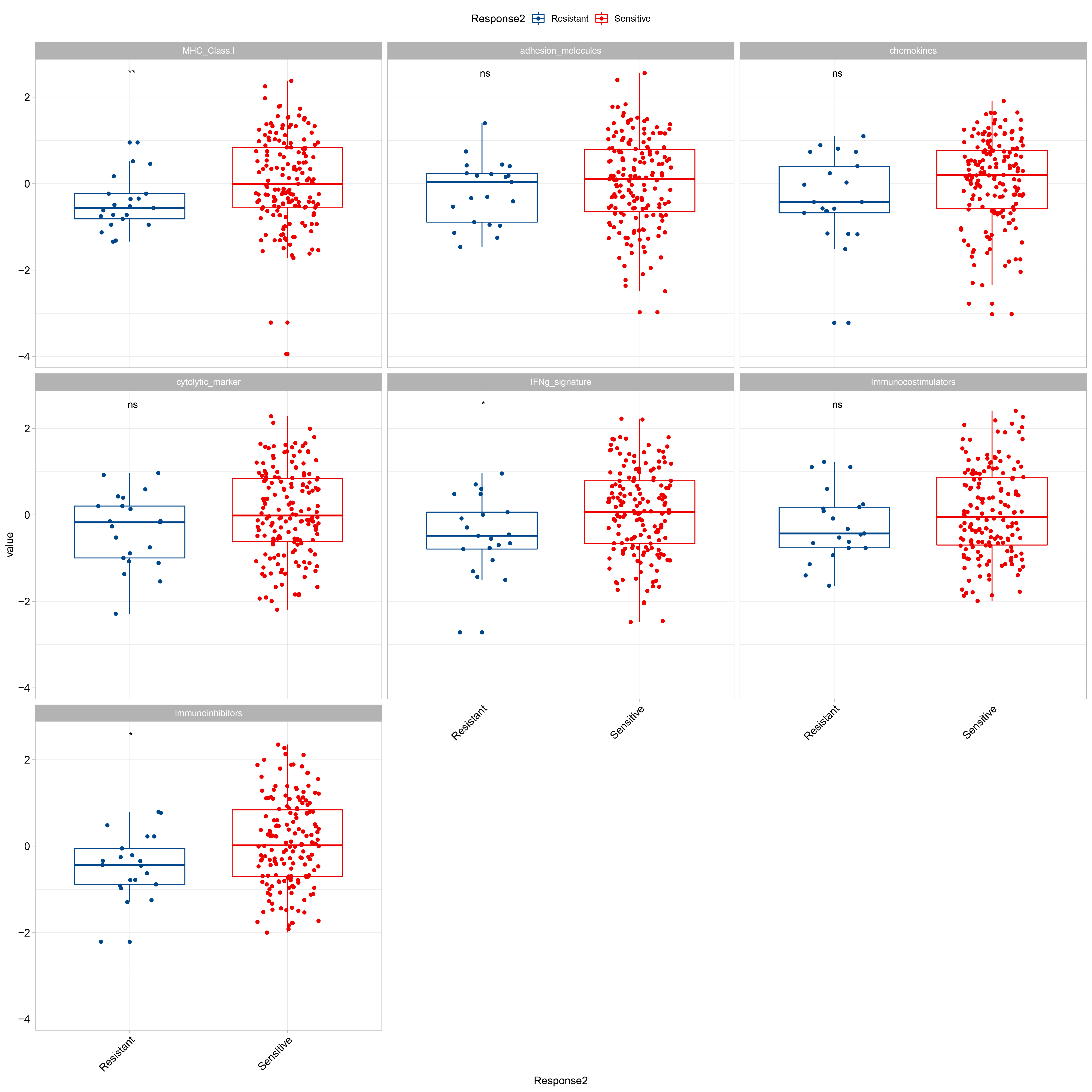

Supplement: Supplementary Figure 2-6 — Comparison of differences in immune characteristics between the drug-resistant group and drug-sensitive group. [file Image_2.jpeg]

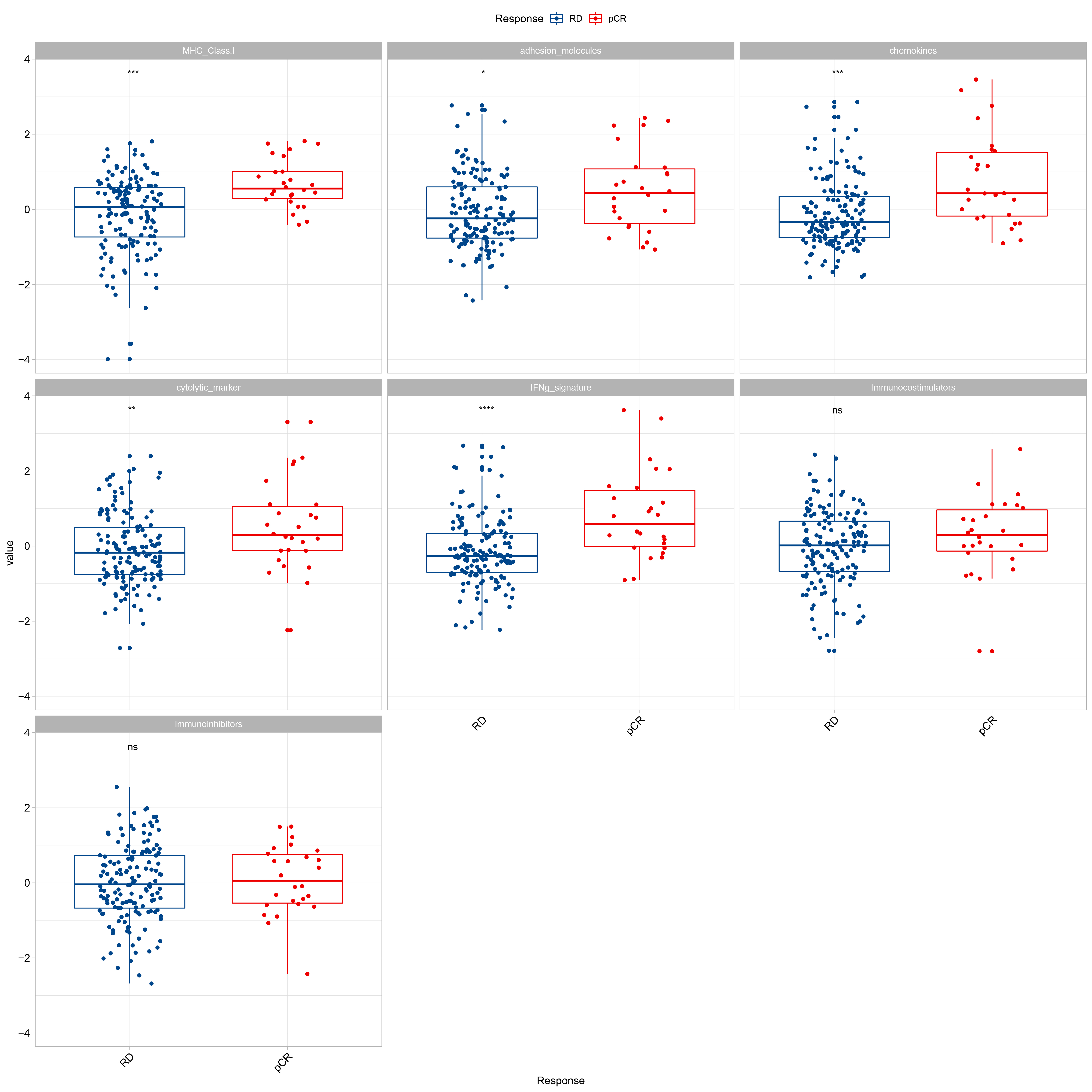

Supplement: Supplementary file 3 [file Image_3.jpeg]

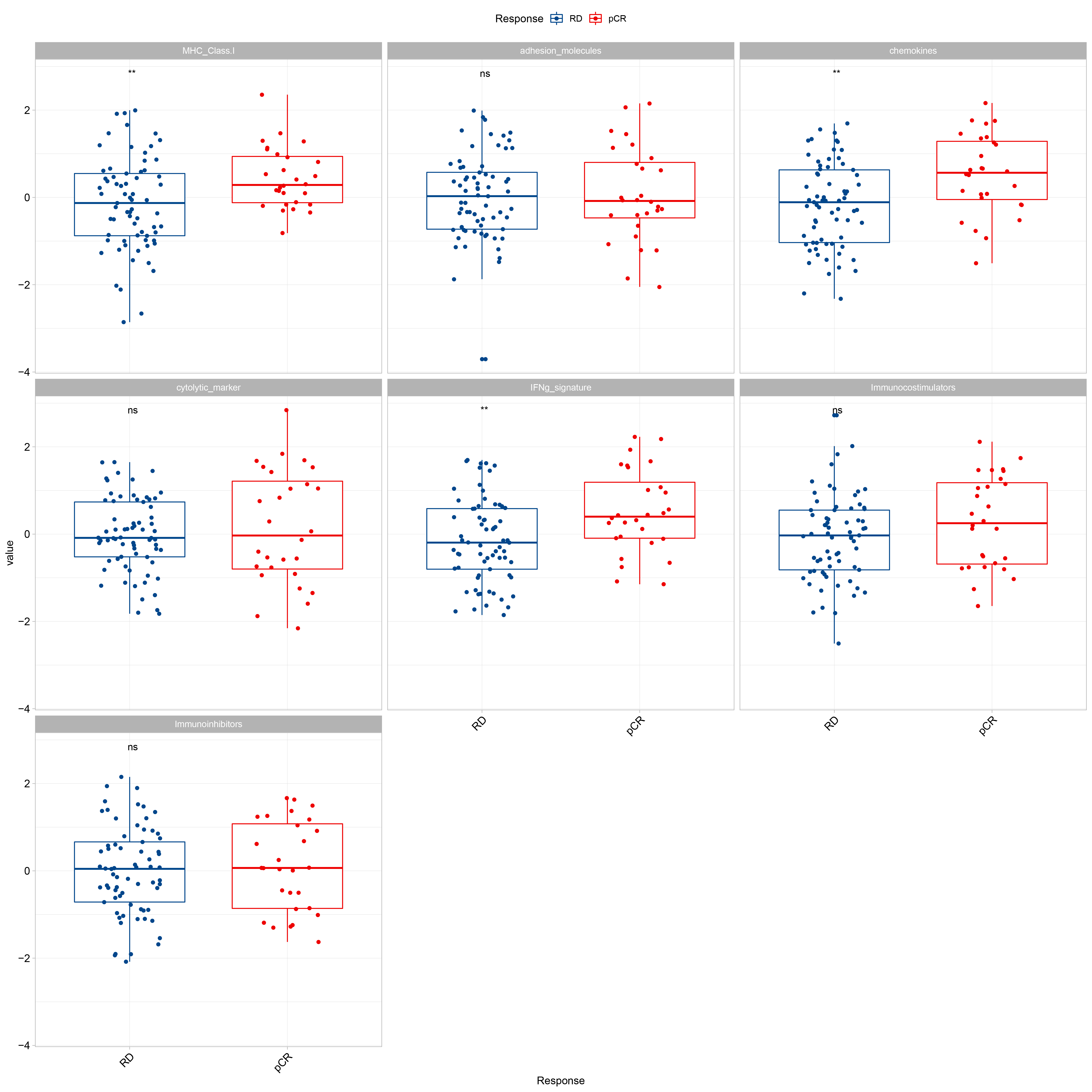

Supplement: Supplementary file 4 [file Image_4.jpeg]

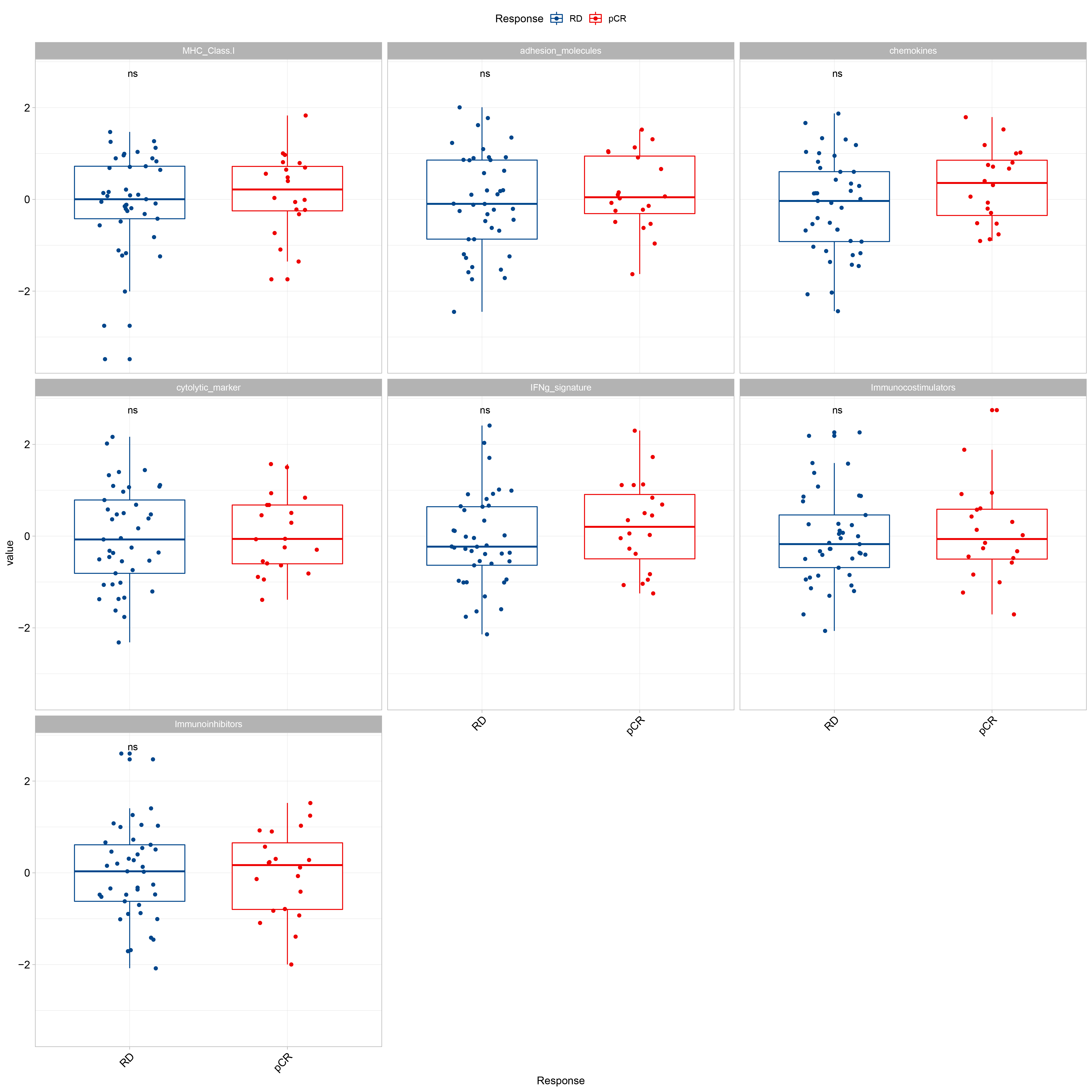

Supplement: Supplementary file 5 [file Image_5.jpeg]

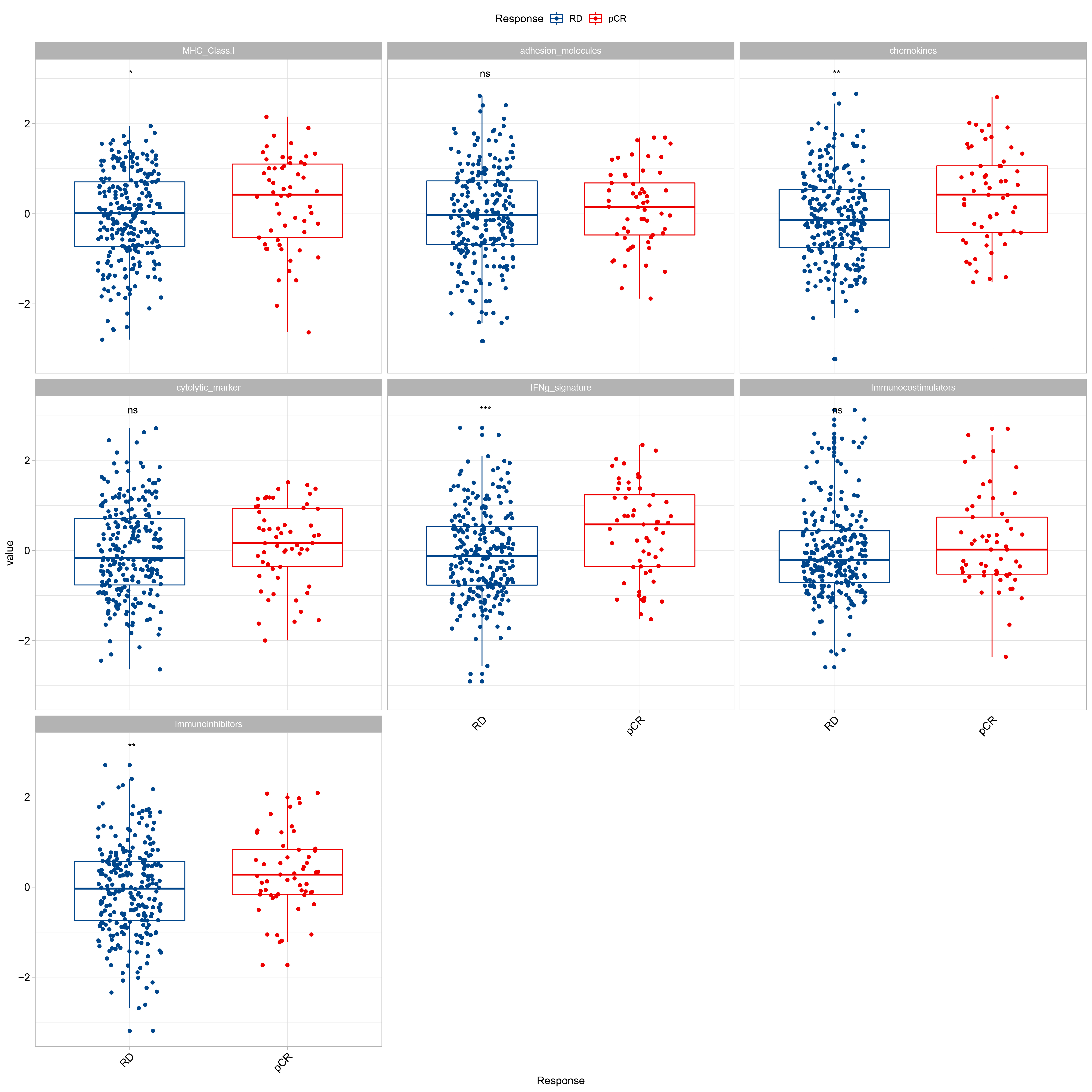

Supplement: Supplementary file 6 [file Image_6.jpeg]
