# Supplementary material for: Identification of Immune-Related Breast Cancer Chemotherapy Resistance Genes via Bioinformatics Approaches
Source: Front Oncol. 2022 Mar 21;12:772723. doi: 10.3389/fonc.2022.772723 (PMC8978268; doi:10.3389/fonc.2022.772723)
Supplement: Supplementary file 7 [file Table_1.docx]

| Gene | logFC | AveExpr | t | P.Value | adj.P.Val | B | diff_label |
| --- | --- | --- | --- | --- | --- | --- | --- |
| EIF4B | -0.60826 | 9.999054 | -4.77848 | 9.02E-05 | 0.695608 | -2.05752 | Down |
| RASSF3 | 1.161919 | 8.439657 | 4.36642 | 0.000247 | 0.97328 | -2.33616 | Up |
| RAI2 | -2.01038 | 6.26512 | -4.21428 | 0.000358 | 0.97328 | -2.4432 | Down |
| CYP27B1 | 1.353914 | 5.255648 | 3.984457 | 0.000626 | 0.97328 | -2.60873 | Up |
| APOL6 | 0.95695 | 6.152513 | 3.848222 | 0.000873 | 0.97328 | -2.70883 | Up |
| FAXC | 0.712762 | 4.181687 | 3.847741 | 0.000874 | 0.97328 | -2.70919 | Up |
| ACE | 0.59875 | 5.084618 | 3.78136 | 0.001026 | 0.97328 | -2.75844 | Up |
| KIF3C | 0.713437 | 6.199953 | 3.767407 | 0.001062 | 0.97328 | -2.76883 | Up |
| HMGCS1 | 0.781656 | 5.694045 | 3.759788 | 0.001082 | 0.97328 | -2.77451 | Up |
| IFIH1 | 0.786555 | 3.668769 | 3.746656 | 0.001117 | 0.97328 | -2.7843 | Up |
| SLC9A3.AS1 | -1.00501 | 5.421795 | -3.72278 | 0.001183 | 0.97328 | -2.80214 | Down |
| C10orf82 | -1.32694 | 4.850877 | -3.70651 | 0.001231 | 0.97328 | -2.81432 | Down |
| CAB39L | -0.88465 | 6.169553 | -3.60776 | 0.001562 | 0.996764 | -2.88855 | Down |
| ERMP1 | -1.02826 | 8.200798 | -3.5989 | 0.001596 | 0.996764 | -2.89523 | Down |
| PEX11A | -1.0384 | 6.898868 | -3.58828 | 0.001637 | 0.996764 | -2.90325 | Down |
| PEG3 | -2.03482 | 4.884876 | -3.57276 | 0.001699 | 0.996764 | -2.91498 | Down |
| SKA1 | 0.61687 | 3.527929 | 3.558467 | 0.001759 | 0.996764 | -2.9258 | Up |
| SPTBN2 | 0.675354 | 6.00681 | 3.5533 | 0.001781 | 0.996764 | -2.92971 | Up |
| ANAPC15 | -0.76498 | 7.49887 | -3.51954 | 0.001931 | 0.996764 | -2.9553 | Down |
| EXO1 | 1.279237 | 5.996914 | 3.511387 | 0.00197 | 0.996764 | -2.96148 | Up |
| ECT2 | 0.706324 | 5.176864 | 3.511227 | 0.00197 | 0.996764 | -2.9616 | Up |
| ZNF879 | -0.89029 | 5.98889 | -3.50203 | 0.002014 | 0.996764 | -2.96859 | Down |
| DONSON | 1.002845 | 6.827869 | 3.491986 | 0.002063 | 0.996764 | -2.97622 | Up |
| EAF1 | 0.634094 | 6.59169 | 3.438142 | 0.002347 | 0.996764 | -3.01719 | Up |
| NCAPH | 0.944901 | 6.165778 | 3.408695 | 0.002519 | 0.996764 | -3.03965 | Up |
| CLUAP1 | -0.64688 | 6.03969 | -3.40659 | 0.002531 | 0.996764 | -3.04125 | Down |
| NNT.AS1 | 0.73692 | 6.432166 | 3.395008 | 0.002602 | 0.996764 | -3.0501 | Up |
| SCLT1 | 0.617528 | 3.95593 | 3.390801 | 0.002628 | 0.996764 | -3.05331 | Up |
| SYT17 | -1.97188 | 6.540362 | -3.37252 | 0.002745 | 0.996764 | -3.06728 | Down |
| OIP5 | 1.015257 | 6.209066 | 3.357082 | 0.002848 | 0.996764 | -3.07908 | Up |
| KPNA2 | 1.023909 | 10.51953 | 3.349498 | 0.0029 | 0.996764 | -3.08488 | Up |
| P2RY2 | -0.83423 | 5.690265 | -3.27559 | 0.003456 | 0.996764 | -3.1415 | Down |
| CDC42EP1 | 0.723861 | 6.927489 | 3.273972 | 0.00347 | 0.996764 | -3.14274 | Up |
| EPCAM | 0.876318 | 11.15853 | 3.273462 | 0.003474 | 0.996764 | -3.14313 | Up |
| SCO2 | 0.725222 | 9.106995 | 3.251816 | 0.003656 | 0.996764 | -3.15974 | Up |
| BLCAP | -0.60572 | 8.758213 | -3.20147 | 0.004118 | 0.996764 | -3.19839 | Down |
| TCF19 | 0.759321 | 6.123147 | 3.199449 | 0.004137 | 0.996764 | -3.19995 | Up |
| GSPT1 | -0.71184 | 7.418456 | -3.19684 | 0.004163 | 0.996764 | -3.20195 | Down |
| PNPT1 | 0.706599 | 8.502035 | 3.181745 | 0.004314 | 0.996764 | -3.21355 | Up |
| LCMT1 | -0.61677 | 8.930983 | -3.16567 | 0.00448 | 0.996764 | -3.22591 | Down |
| CDCA8 | 0.996133 | 7.097623 | 3.148666 | 0.004662 | 0.996764 | -3.23898 | Up |
| PATL1 | 0.588841 | 5.99205 | 3.141861 | 0.004737 | 0.996764 | -3.24421 | Up |
| CKAP2L | 0.786546 | 6.23725 | 3.13359 | 0.00483 | 0.996764 | -3.25057 | Up |
| WIPI1 | 0.623633 | 8.03362 | 3.123681 | 0.004944 | 0.996764 | -3.25819 | Up |
| ACSF2 | -0.65506 | 8.042614 | -3.1027 | 0.005192 | 0.996764 | -3.27433 | Down |
| KDM1B | 0.688357 | 4.788318 | 3.101933 | 0.005202 | 0.996764 | -3.27492 | Up |
| FCGBP | -1.31619 | 6.5965 | -3.09234 | 0.00532 | 0.996764 | -3.28229 | Down |
| FGD1 | 0.764551 | 6.481832 | 3.040522 | 0.006003 | 0.996764 | -3.32214 | Up |
| NLRP2 | -1.05338 | 5.057087 | -3.03567 | 0.006071 | 0.996764 | -3.32588 | Down |
| IFRD1 | 0.741002 | 6.886617 | 3.03174 | 0.006126 | 0.996764 | -3.3289 | Up |
| CX3CR1 | -0.86292 | 5.539698 | -3.02951 | 0.006158 | 0.996764 | -3.33061 | Down |
| BMERB1 | -0.64001 | 6.650448 | -3.01496 | 0.00637 | 0.996764 | -3.3418 | Down |
| IRF9 | 0.713749 | 9.350046 | 3.013895 | 0.006386 | 0.996764 | -3.34262 | Up |
| TPTEP1 | -0.65684 | 4.666314 | -2.99977 | 0.006598 | 0.996764 | -3.35348 | Down |
| SV2A | 0.638019 | 4.970303 | 2.986214 | 0.006809 | 0.996764 | -3.3639 | Up |
| SMURF2 | 0.628405 | 7.008607 | 2.97776 | 0.006943 | 0.996764 | -3.37039 | Up |
| EZH2 | 1.051545 | 6.909313 | 2.963802 | 0.007171 | 0.996764 | -3.38112 | Up |
| SMC6 | 0.598053 | 6.338849 | 2.946581 | 0.007461 | 0.996764 | -3.39434 | Up |
| PWWP3B | -1.68204 | 5.853189 | -2.9146 | 0.008031 | 0.996764 | -3.41889 | Down |
| HSPA2 | -1.85993 | 7.393561 | -2.91183 | 0.008083 | 0.996764 | -3.42102 | Down |
| STAT1 | 1.294812 | 8.57849 | 2.910807 | 0.008102 | 0.996764 | -3.42181 | Up |
| BRIP1 | 1.116286 | 4.933926 | 2.901812 | 0.008271 | 0.996764 | -3.42871 | Up |
| SECTM1 | 0.961825 | 8.045781 | 2.890746 | 0.008484 | 0.996764 | -3.43719 | Up |
| E2F8 | 1.12897 | 5.368222 | 2.888917 | 0.008519 | 0.996764 | -3.4386 | Up |
| ENOSF1 | -0.6893 | 7.302425 | -2.88611 | 0.008574 | 0.996764 | -3.44075 | Down |
| IL6ST | -1.32873 | 7.009075 | -2.86895 | 0.008918 | 0.996764 | -3.4539 | Down |
| TMEM99 | -1.05393 | 8.464379 | -2.84703 | 0.009376 | 0.996764 | -3.47069 | Down |
| CENPL | 0.709665 | 6.091448 | 2.84311 | 0.00946 | 0.996764 | -3.47369 | Up |
| CHAF1B | 0.610204 | 6.116074 | 2.8336 | 0.009668 | 0.996764 | -3.48097 | Up |
| OAS3 | 0.636486 | 6.023417 | 2.806084 | 0.010292 | 0.996764 | -3.50201 | Up |
| RRM2 | 1.141802 | 9.015289 | 2.805857 | 0.010298 | 0.996764 | -3.50219 | Up |
| ALKBH3 | -0.87443 | 7.210071 | -2.80474 | 0.010324 | 0.996764 | -3.50304 | Down |
| CCL8 | 1.834113 | 7.25288 | 2.789917 | 0.010677 | 0.996764 | -3.51436 | Up |
| EPB41L4A.AS1 | -0.71801 | 8.80664 | -2.78092 | 0.010897 | 0.996764 | -3.52124 | Down |
| SMC4 | 0.690837 | 8.036248 | 2.779486 | 0.010933 | 0.996764 | -3.52233 | Up |
| CDCA5 | 0.867096 | 7.256966 | 2.766161 | 0.011267 | 0.996764 | -3.53249 | Up |
| PSMD12 | 0.763615 | 8.713831 | 2.761337 | 0.011391 | 0.996764 | -3.53617 | Up |
| GPSM2 | 0.722919 | 6.242199 | 2.756889 | 0.011506 | 0.996764 | -3.53956 | Up |
| IP6K2 | -0.58605 | 7.894903 | -2.75543 | 0.011544 | 0.996764 | -3.54067 | Down |
| CYFIP2 | -0.9451 | 7.169025 | -2.74291 | 0.011875 | 0.996764 | -3.55022 | Down |
| LIN28A | 1.33568 | 3.551674 | 2.729968 | 0.012227 | 0.996764 | -3.56007 | Up |
| DBF4 | 0.746073 | 7.838323 | 2.728578 | 0.012265 | 0.996764 | -3.56113 | Up |
| GGTLC1 | 0.858684 | 6.326962 | 2.697167 | 0.013162 | 0.996764 | -3.58501 | Up |
| KLHDC9 | -0.86291 | 8.206224 | -2.69124 | 0.013338 | 0.996764 | -3.58951 | Down |
| SNHG19 | -0.94669 | 9.406583 | -2.68572 | 0.013504 | 0.996764 | -3.5937 | Down |
| LMNB1 | 0.762506 | 6.292959 | 2.671505 | 0.013941 | 0.996764 | -3.60448 | Up |
| ADRA2A | -1.07319 | 6.639396 | -2.67031 | 0.013978 | 0.996764 | -3.60539 | Down |
| UBE2S | 0.838011 | 8.409153 | 2.663684 | 0.014187 | 0.996764 | -3.61041 | Up |
| LOC100287896 | -0.78882 | 5.56435 | -2.654 | 0.014497 | 0.996764 | -3.61774 | Down |
| LOC105374809 | -0.69958 | 5.003191 | -2.65054 | 0.014609 | 0.996764 | -3.62036 | Down |
| VTCN1 | -1.69419 | 8.210725 | -2.64316 | 0.014851 | 0.996764 | -3.62595 | Down |
| RAP2C | 0.69072 | 8.121138 | 2.639487 | 0.014973 | 0.996764 | -3.62872 | Up |
| MAP3K1 | -0.69473 | 7.231242 | -2.63865 | 0.015001 | 0.996764 | -3.62936 | Down |
| NCAPG2 | 0.651938 | 6.653712 | 2.636593 | 0.01507 | 0.996764 | -3.63091 | Up |
| GSTM3 | -1.2494 | 8.228063 | -2.63226 | 0.015216 | 0.996764 | -3.63419 | Down |
| DEPDC1 | 0.943731 | 5.209279 | 2.631219 | 0.015251 | 0.996764 | -3.63498 | Up |
| KIF4A | 0.994893 | 6.764458 | 2.627664 | 0.015372 | 0.996764 | -3.63766 | Up |
| ZNF542P | -0.76284 | 6.017477 | -2.62124 | 0.015593 | 0.996764 | -3.64251 | Down |
| NTN4 | -1.39598 | 8.14008 | -2.61772 | 0.015716 | 0.996764 | -3.64517 | Down |
| ADIRF | -1.58859 | 10.41407 | -2.61031 | 0.015976 | 0.996764 | -3.65076 | Down |
| SLC44A1 | -0.65483 | 6.460421 | -2.60164 | 0.016286 | 0.996764 | -3.6573 | Down |
| KCNJ3 | 1.311552 | 3.763379 | 2.601341 | 0.016297 | 0.996764 | -3.65753 | Up |
| CDC45 | 0.775338 | 6.408365 | 2.598729 | 0.016392 | 0.996764 | -3.6595 | Up |
| GSTM2 | -1.16507 | 8.076919 | -2.59849 | 0.0164 | 0.996764 | -3.65968 | Down |
| CCNE2 | 1.158101 | 5.968179 | 2.586539 | 0.01684 | 0.996764 | -3.66868 | Up |
| ARL9 | 0.931821 | 5.375373 | 2.586451 | 0.016843 | 0.996764 | -3.66874 | Up |
| PGR | -2.09959 | 5.870249 | -2.58583 | 0.016866 | 0.996764 | -3.66921 | Down |
| PTGER3 | -0.84416 | 4.773111 | -2.5814 | 0.017032 | 0.996764 | -3.67255 | Down |
| CNTN4 | -0.73742 | 4.406449 | -2.57817 | 0.017154 | 0.996764 | -3.67498 | Down |
| CASC1 | -0.79442 | 3.634972 | -2.57744 | 0.017182 | 0.996764 | -3.67552 | Down |
| SLC25A33 | 0.741798 | 7.971968 | 2.571327 | 0.017415 | 0.996764 | -3.68012 | Up |
| LOC100507395 | -0.59693 | 4.53006 | -2.56111 | 0.017812 | 0.996764 | -3.6878 | Down |
| CDT1 | 0.824291 | 6.238061 | 2.556763 | 0.017983 | 0.996764 | -3.69106 | Up |
| FRMD6 | -0.73926 | 9.070846 | -2.55592 | 0.018017 | 0.996764 | -3.69169 | Down |
| KIF18B | 0.792678 | 6.723675 | 2.544798 | 0.018463 | 0.996764 | -3.70004 | Up |
| B9D1 | -0.71441 | 5.930147 | -2.5337 | 0.018919 | 0.996764 | -3.70836 | Down |
| CMPK2 | 1.47231 | 8.093305 | 2.5327 | 0.01896 | 0.996764 | -3.7091 | Up |
| COL9A2 | 0.719043 | 6.053076 | 2.532489 | 0.018969 | 0.996764 | -3.70926 | Up |
| TENT5B | -1.02543 | 7.455874 | -2.53244 | 0.018971 | 0.996764 | -3.7093 | Down |
| KAT2B | 0.718444 | 6.354887 | 2.528229 | 0.019147 | 0.996764 | -3.71245 | Up |
| PTPRM | -0.62216 | 7.435032 | -2.52634 | 0.019227 | 0.996764 | -3.71387 | Down |
| MCC | -0.66558 | 6.137464 | -2.5221 | 0.019406 | 0.996764 | -3.71704 | Down |
| MXI1 | -0.7249 | 9.735205 | -2.52165 | 0.019425 | 0.996764 | -3.71738 | Down |
| ZBTB18 | -1.03393 | 6.094154 | -2.51469 | 0.019724 | 0.996764 | -3.72258 | Down |
| SPAG5 | 0.993742 | 7.642679 | 2.512098 | 0.019836 | 0.996764 | -3.72452 | Up |
| BUB1B | 0.955604 | 7.578722 | 2.511649 | 0.019855 | 0.996764 | -3.72485 | Up |
| RAB26 | -1.12512 | 6.919163 | -2.50666 | 0.020073 | 0.996764 | -3.72858 | Down |
| SLC5A11 | 0.638392 | 4.75609 | 2.500143 | 0.020361 | 0.996764 | -3.73344 | Up |
| AGPS | 0.792109 | 6.288465 | 2.498935 | 0.020415 | 0.996764 | -3.73435 | Up |
| FBXO6 | 0.748268 | 8.006428 | 2.496038 | 0.020544 | 0.996764 | -3.73651 | Up |
| CRIP2 | -0.81337 | 8.725599 | -2.49134 | 0.020756 | 0.996764 | -3.74001 | Down |
| PTTG1 | 0.730005 | 9.427746 | 2.490565 | 0.020791 | 0.996764 | -3.74059 | Up |
| GNA13 | 0.684553 | 7.359025 | 2.482829 | 0.021145 | 0.996764 | -3.74635 | Up |
| NME3 | -0.7331 | 8.791381 | -2.47581 | 0.02147 | 0.996764 | -3.75158 | Down |
| MX1 | 1.383896 | 9.852331 | 2.470732 | 0.021709 | 0.996764 | -3.75536 | Up |
| ELOVL2 | -1.45288 | 5.361992 | -2.45814 | 0.02231 | 0.996764 | -3.76471 | Down |
| SPC25 | 0.866149 | 5.325525 | 2.452883 | 0.022566 | 0.996764 | -3.76862 | Up |
| TDRP | -0.7996 | 5.42768 | -2.44879 | 0.022767 | 0.996764 | -3.77165 | Down |
| KNSTRN | 0.649222 | 7.308046 | 2.448702 | 0.022772 | 0.996764 | -3.77172 | Up |
| LINC00922 | -0.5946 | 4.650403 | -2.44644 | 0.022884 | 0.996764 | -3.7734 | Down |
| PRC1 | 1.004424 | 8.208206 | 2.44485 | 0.022963 | 0.996764 | -3.77458 | Up |
| ELOVL2.AS1 | -0.9514 | 3.647389 | -2.44203 | 0.023103 | 0.996764 | -3.77667 | Down |
| LURAP1L | -0.99388 | 7.991433 | -2.43857 | 0.023277 | 0.996764 | -3.77923 | Down |
| KLHDC7B | 1.722849 | 6.794668 | 2.433831 | 0.023517 | 0.996764 | -3.78274 | Up |
| CT83 | 1.598688 | 3.689507 | 2.430173 | 0.023703 | 0.996764 | -3.78545 | Up |
| CEACAM5 | 0.930074 | 5.323112 | 2.428724 | 0.023778 | 0.996764 | -3.78652 | Up |
| MNX1.AS1 | 0.873845 | 4.125567 | 2.428036 | 0.023813 | 0.996764 | -3.78703 | Up |
| MARCKS | 0.702316 | 9.168878 | 2.427985 | 0.023816 | 0.996764 | -3.78707 | Up |
| TMEM101 | -0.63381 | 7.571206 | -2.42141 | 0.024156 | 0.996764 | -3.79193 | Down |
| MT1F | 0.621195 | 8.652862 | 2.419475 | 0.024257 | 0.996764 | -3.79336 | Up |
| MPV17L | -0.77118 | 5.991525 | -2.41944 | 0.024259 | 0.996764 | -3.79339 | Down |
| KIF1B | 0.594437 | 6.891512 | 2.417799 | 0.024345 | 0.996764 | -3.7946 | Up |
| GCNT1 | 0.626017 | 5.66142 | 2.41749 | 0.024361 | 0.996764 | -3.79483 | Up |
| USP18 | 0.927685 | 7.159051 | 2.411731 | 0.024665 | 0.996764 | -3.79908 | Up |
| KIF2C | 0.868301 | 6.973248 | 2.410167 | 0.024749 | 0.996764 | -3.80023 | Up |
| ARL5B | 0.639863 | 6.078725 | 2.402367 | 0.025168 | 0.996764 | -3.80599 | Up |
| C16orf89 | -1.6357 | 6.331669 | -2.39703 | 0.025458 | 0.996764 | -3.80992 | Down |
| METTL7B | 0.690732 | 4.67424 | 2.396898 | 0.025465 | 0.996764 | -3.81002 | Up |
| GZMB | 1.595107 | 6.449892 | 2.391345 | 0.025771 | 0.996764 | -3.81411 | Up |
| PPP3CA | -0.60341 | 8.431556 | -2.391 | 0.02579 | 0.996764 | -3.81437 | Down |
| ITPRIPL2 | -0.7593 | 7.858035 | -2.38834 | 0.025938 | 0.996764 | -3.81632 | Down |
| SKA2 | 0.806115 | 8.092389 | 2.387364 | 0.025992 | 0.996764 | -3.81704 | Up |
| MTHFD2 | 0.644516 | 10.32251 | 2.383568 | 0.026205 | 0.996764 | -3.81983 | Up |
| PANK1 | 0.753768 | 5.890605 | 2.383191 | 0.026226 | 0.996764 | -3.82011 | Up |
| SLC7A8 | -1.04359 | 7.087587 | -2.3828 | 0.026248 | 0.996764 | -3.8204 | Down |
| TAP2 | 0.761974 | 5.686535 | 2.377755 | 0.026533 | 0.996764 | -3.82411 | Up |
| GPALPP1 | -0.61431 | 6.201678 | -2.3768 | 0.026587 | 0.996764 | -3.82481 | Down |
| PITX1 | 0.757244 | 5.886592 | 2.375476 | 0.026663 | 0.996764 | -3.82578 | Up |
| LRRC17 | -0.83177 | 5.164816 | -2.37033 | 0.026958 | 0.996764 | -3.82956 | Down |
| CLDN10 | 0.867674 | 3.873114 | 2.365649 | 0.02723 | 0.996764 | -3.833 | Up |
| RPS15A | -0.59517 | 6.709451 | -2.361 | 0.027501 | 0.996764 | -3.8364 | Down |
| WASF3 | -0.83729 | 6.408773 | -2.35774 | 0.027694 | 0.996764 | -3.83879 | Down |
| LGMN | 0.609075 | 9.476149 | 2.345888 | 0.028403 | 0.996764 | -3.84747 | Up |
| GSTM1 | -0.99997 | 7.597987 | -2.3407 | 0.028719 | 0.996764 | -3.85127 | Down |
| ARHGDIB | -0.6506 | 9.62166 | -2.33209 | 0.02925 | 0.996764 | -3.85756 | Down |
| ZNF677 | -0.60653 | 4.373337 | -2.32337 | 0.029796 | 0.996764 | -3.86392 | Down |
| S100A14 | -1.05654 | 9.274655 | -2.32289 | 0.029827 | 0.996764 | -3.86427 | Down |
| BLM | 0.645732 | 6.10905 | 2.320458 | 0.029981 | 0.996764 | -3.86604 | Up |
| HPSE | 0.653341 | 4.611794 | 2.318856 | 0.030083 | 0.996764 | -3.86721 | Up |
| TYMP | 0.674772 | 7.037347 | 2.315308 | 0.03031 | 0.996764 | -3.86979 | Up |
| FAM155B | 0.609657 | 4.593534 | 2.315253 | 0.030314 | 0.996764 | -3.86983 | Up |
| DHTKD1 | 0.901261 | 8.13795 | 2.313968 | 0.030397 | 0.996764 | -3.87077 | Up |
| LGALS3BP | 0.947729 | 10.9877 | 2.313287 | 0.030441 | 0.996764 | -3.87126 | Up |
| KIF18A | 0.834439 | 5.003863 | 2.308926 | 0.030723 | 0.996764 | -3.87444 | Up |
| DNAJC1 | 0.788659 | 9.781394 | 2.307438 | 0.03082 | 0.996764 | -3.87552 | Up |
| ZNF595 | 0.715405 | 3.720328 | 2.303836 | 0.031056 | 0.996764 | -3.87814 | Up |
| ZNF367 | 0.943193 | 5.903944 | 2.299135 | 0.031366 | 0.996764 | -3.88155 | Up |
| TP53I13 | 0.735698 | 6.945132 | 2.29702 | 0.031507 | 0.996764 | -3.88309 | Up |
| STIL | 0.823911 | 6.721808 | 2.291684 | 0.031864 | 0.996764 | -3.88696 | Up |
| IRF6 | 0.672664 | 7.197555 | 2.284327 | 0.032362 | 0.996764 | -3.89229 | Up |
| PADI3 | 0.696188 | 4.349195 | 2.282288 | 0.032502 | 0.996764 | -3.89377 | Up |
| TAP1 | 0.964474 | 8.984513 | 2.279482 | 0.032694 | 0.996764 | -3.8958 | Up |
| CLIC3 | 1.003805 | 5.95612 | 2.276986 | 0.032867 | 0.996764 | -3.89761 | Up |
| WARS | 0.762888 | 8.90424 | 2.263046 | 0.033844 | 0.996764 | -3.90768 | Up |
| PLSCR1 | 0.716845 | 7.756626 | 2.252542 | 0.034599 | 0.996764 | -3.91526 | Up |
| PARP12 | 0.816566 | 8.665895 | 2.251396 | 0.034682 | 0.996764 | -3.91609 | Up |
| AURKA | 0.836888 | 7.496955 | 2.249007 | 0.034856 | 0.996764 | -3.91781 | Up |
| PREX1 | -0.84297 | 8.685684 | -2.24853 | 0.034891 | 0.996764 | -3.91815 | Down |
| APOBEC3B | 1.403688 | 6.834318 | 2.245284 | 0.035129 | 0.996764 | -3.92049 | Up |
| MPP7 | -0.64436 | 4.905037 | -2.24328 | 0.035276 | 0.996764 | -3.92193 | Down |
| BIRC5 | 0.879463 | 6.171068 | 2.241749 | 0.03539 | 0.996764 | -3.92303 | Up |
| MAGEA6 | 1.314416 | 3.391133 | 2.241441 | 0.035412 | 0.996764 | -3.92325 | Up |
| C1QC | 0.752854 | 8.988551 | 2.236797 | 0.035758 | 0.996764 | -3.92659 | Up |
| TSTD1 | -0.65643 | 10.31799 | -2.23542 | 0.035861 | 0.996764 | -3.92758 | Down |
| HORMAD1 | 1.335695 | 3.549369 | 2.233536 | 0.036003 | 0.996764 | -3.92893 | Up |
| EBPL | -0.60371 | 9.23726 | -2.23241 | 0.036087 | 0.996764 | -3.92974 | Down |
| GABRB3 | 0.806796 | 3.5761 | 2.23041 | 0.036238 | 0.996764 | -3.93118 | Up |
| CEP55 | 0.940766 | 6.452555 | 2.224906 | 0.036657 | 0.996764 | -3.93513 | Up |
| C1QB | 1.018394 | 9.343106 | 2.219766 | 0.037052 | 0.996764 | -3.93881 | Up |
| FAM83B | 0.756586 | 4.4332 | 2.212034 | 0.037653 | 0.996764 | -3.94435 | Up |
| NPY1R | -2.26339 | 7.319402 | -2.20919 | 0.037877 | 0.996764 | -3.94638 | Down |
| PLAUR | 0.664429 | 7.938428 | 2.207983 | 0.037972 | 0.996764 | -3.94724 | Up |
| PIK3R1 | -0.63753 | 8.091732 | -2.2075 | 0.03801 | 0.996764 | -3.94759 | Down |
| ADAMDEC1 | 1.817299 | 7.166436 | 2.205906 | 0.038136 | 0.996764 | -3.94873 | Up |
| CCNG2 | -0.68647 | 8.148477 | -2.19458 | 0.039044 | 0.996764 | -3.95681 | Down |
| LYZ | 1.453387 | 9.124022 | 2.184695 | 0.039851 | 0.996764 | -3.96385 | Up |
| MEIS3P1 | -0.60378 | 7.576329 | -2.18295 | 0.039995 | 0.996764 | -3.96509 | Down |
| EVA1B | -0.75573 | 8.134983 | -2.18274 | 0.040013 | 0.996764 | -3.96524 | Down |
| CCL7 | 0.834501 | 3.942745 | 2.179841 | 0.040253 | 0.996764 | -3.96731 | Up |
| GBP5 | 1.136241 | 6.092808 | 2.176813 | 0.040506 | 0.996764 | -3.96946 | Up |
| MSX2 | -0.84219 | 5.676437 | -2.17469 | 0.040684 | 0.996764 | -3.97097 | Down |
| FLNB | -0.7808 | 9.092597 | -2.16101 | 0.041849 | 0.996764 | -3.98067 | Down |
| KMO | 1.400074 | 6.833541 | 2.160915 | 0.041857 | 0.996764 | -3.98074 | Up |
| IRS1 | -0.66667 | 5.875717 | -2.15874 | 0.042045 | 0.996764 | -3.98228 | Down |
| KLRG2 | 0.770789 | 5.742116 | 2.15531 | 0.042343 | 0.996764 | -3.98471 | Up |
| ARRB1 | -0.62427 | 7.086334 | -2.15257 | 0.042582 | 0.996764 | -3.98664 | Down |
| KIF11 | 0.935949 | 6.412125 | 2.147664 | 0.043014 | 0.996764 | -3.99011 | Up |
| DSP | -1.39182 | 11.03011 | -2.14716 | 0.043058 | 0.996764 | -3.99047 | Down |
| CLIC6 | -1.49493 | 5.40636 | -2.14164 | 0.043549 | 0.996764 | -3.99436 | Down |
| MUC15 | 0.857484 | 3.682732 | 2.13733 | 0.043936 | 0.996764 | -3.9974 | Up |
| CD38 | 1.067433 | 5.686425 | 2.137185 | 0.043949 | 0.996764 | -3.99751 | Up |
| LINC01208 | 0.641212 | 5.56508 | 2.123417 | 0.045206 | 0.996764 | -4.0072 | Up |
| APOL3 | 0.715687 | 7.511759 | 2.121778 | 0.045358 | 0.996764 | -4.00835 | Up |
| KDELR3 | 0.659689 | 7.294042 | 2.12155 | 0.045379 | 0.996764 | -4.00851 | Up |
| BEX1 | -1.48767 | 5.450997 | -2.12039 | 0.045487 | 0.996764 | -4.00933 | Down |
| MIR3682 | 0.604184 | 4.393198 | 2.115749 | 0.04592 | 0.996764 | -4.01259 | Up |
| DTL | 0.862612 | 7.251988 | 2.115168 | 0.045974 | 0.996764 | -4.01299 | Up |
| ADGRG7 | 1.19754 | 3.330168 | 2.113789 | 0.046104 | 0.996764 | -4.01396 | Up |
| PPM1D | 0.623602 | 6.124221 | 2.109406 | 0.046518 | 0.996764 | -4.01703 | Up |
| NDP | -1.25159 | 4.124319 | -2.10778 | 0.046672 | 0.996764 | -4.01817 | Down |
| SPAG1 | 0.761038 | 6.244779 | 2.107153 | 0.046732 | 0.996764 | -4.01861 | Up |
| CENPN | 0.720331 | 5.654626 | 2.105634 | 0.046877 | 0.996764 | -4.01968 | Up |
| ERAP2 | 0.767188 | 4.566435 | 2.103692 | 0.047063 | 0.996764 | -4.02104 | Up |
| MCM10 | 0.954418 | 5.395544 | 2.103107 | 0.047119 | 0.996764 | -4.02144 | Up |
| CXCL12 | -0.76468 | 9.089821 | -2.10109 | 0.047313 | 0.996764 | -4.02285 | Down |
| RNF19B | 0.603423 | 7.7775 | 2.099627 | 0.047454 | 0.996764 | -4.02388 | Up |
| PRAME | 1.634871 | 5.585709 | 2.094564 | 0.047945 | 0.996764 | -4.02742 | Up |
| TRIM59 | 0.585315 | 5.864923 | 2.093796 | 0.04802 | 0.996764 | -4.02795 | Up |
| SUSD3 | -0.80317 | 6.100799 | -2.08683 | 0.048704 | 0.996764 | -4.03282 | Down |
| GSTM4 | -0.68539 | 5.50316 | -2.08382 | 0.049002 | 0.996764 | -4.03491 | Down |
| ACSS3 | 0.777571 | 5.651407 | 2.083466 | 0.049038 | 0.996764 | -4.03516 | Up |
| KL | -1.12331 | 3.659463 | -2.08283 | 0.049101 | 0.996764 | -4.03561 | Down |
| ASCC2 | 0.664782 | 7.689845 | 2.080766 | 0.049307 | 0.996764 | -4.03704 | Up |
| DNAJC12 | -1.59952 | 6.663669 | -2.07954 | 0.04943 | 0.996764 | -4.0379 | Down |
| BRINP3 | 1.285676 | 3.581036 | 2.075615 | 0.049824 | 0.996764 | -4.04063 | Up |
