# Supplementary material for: Identification of Immune-Related Breast Cancer Chemotherapy Resistance Genes via Bioinformatics Approaches
Source: Front Oncol. 2022 Mar 21;12:772723. doi: 10.3389/fonc.2022.772723 (PMC8978268; doi:10.3389/fonc.2022.772723)
Supplement: Supplementary file 8 [file Table_2.docx]

| Gene | logFC | AveExpr | t | P.Value | adj.P.Val | B | diff_label |
| --- | --- | --- | --- | --- | --- | --- | --- |
| RRM2 | 0.773825 | 3.125562 | 17.8448 | 6.94E-11 | 1.40E-06 | 14.15605 | Up |
| HMMR | 0.747488 | 2.758236 | 14.52245 | 1.02E-09 | 9.48E-06 | 12.08998 | Up |
| NUSAP1 | 0.738712 | 2.912182 | 14.16801 | 1.41E-09 | 9.48E-06 | 11.83076 | Up |
| UBE2C | 0.602627 | 3.179235 | 13.03565 | 4.10E-09 | 1.58E-05 | 10.94284 | Up |
| TOP2A | 0.892696 | 2.866684 | 12.90064 | 4.68E-09 | 1.58E-05 | 10.83051 | Up |
| AURKA | 0.665852 | 2.916144 | 11.89238 | 1.31E-08 | 2.65E-05 | 9.944138 | Up |
| CEP55 | 0.781891 | 2.701224 | 11.8027 | 1.44E-08 | 2.65E-05 | 9.861041 | Up |
| TPX2 | 0.682091 | 2.970819 | 11.28719 | 2.52E-08 | 3.92E-05 | 9.369001 | Up |
| BUB1B | 0.675981 | 2.934727 | 10.90417 | 3.88E-08 | 5.22E-05 | 8.986952 | Up |
| EZH2 | 0.740931 | 2.810172 | 10.57752 | 5.64E-08 | 6.47E-05 | 8.64947 | Up |
| KIF4A | 0.743927 | 2.776792 | 10.56881 | 5.70E-08 | 6.47E-05 | 8.640325 | Up |
| ANLN | 0.773724 | 2.675665 | 10.52197 | 6.02E-08 | 6.47E-05 | 8.590981 | Up |
| OIP5 | 0.642318 | 2.72147 | 10.50985 | 6.11E-08 | 6.47E-05 | 8.578176 | Up |
| MELK | 0.692316 | 3.034144 | 10.46873 | 6.41E-08 | 6.47E-05 | 8.534619 | Up |
| KIF20A | 0.699796 | 2.817732 | 10.15949 | 9.25E-08 | 8.49E-05 | 8.201255 | Up |
| CDK1 | 0.63528 | 2.907349 | 9.880237 | 1.30E-07 | 0.000109 | 7.891193 | Up |
| UHRF1 | 0.710975 | 2.969892 | 9.7155 | 1.59E-07 | 0.000124 | 7.704154 | Up |
| PRC1 | 0.645227 | 3.045878 | 9.420541 | 2.31E-07 | 0.000161 | 7.361389 | Up |
| UBE2T | 0.617231 | 2.997603 | 9.209929 | 3.02E-07 | 0.000195 | 7.110299 | Up |
| CDC20 | 0.755515 | 2.957021 | 9.194379 | 3.09E-07 | 0.000195 | 7.091546 | Up |
| CAVIN2 | -0.67429 | 2.622631 | -9.04018 | 3.77E-07 | 0.000231 | 6.903979 | Down |
| CCNE2 | 0.703044 | 2.666384 | 8.93033 | 4.36E-07 | 0.000252 | 6.768558 | Up |
| FAM83D | 0.640745 | 2.954791 | 8.719774 | 5.78E-07 | 0.000315 | 6.504715 | Up |
| LYVE1 | -0.69306 | 2.514302 | -8.43108 | 8.57E-07 | 0.000393 | 6.133647 | Down |
| SPC25 | 0.6433 | 2.523553 | 8.126822 | 1.31E-06 | 0.000563 | 5.730577 | Up |
| DLGAP5 | 0.771173 | 2.727767 | 7.985787 | 1.60E-06 | 0.000607 | 5.539468 | Up |
| NCAPG | 0.678257 | 2.69841 | 7.968208 | 1.64E-06 | 0.000607 | 5.515456 | Up |
| KIF11 | 0.755261 | 2.698298 | 7.963725 | 1.65E-06 | 0.000607 | 5.509326 | Up |
| DTL | 0.641455 | 2.883151 | 7.865046 | 1.91E-06 | 0.000663 | 5.373671 | Up |
| DEPDC1 | 0.659733 | 2.501669 | 7.789336 | 2.13E-06 | 0.0007 | 5.268669 | Up |
| TTK | 0.80216 | 2.735025 | 7.781664 | 2.15E-06 | 0.0007 | 5.257984 | Up |
| KIF15 | 0.613681 | 2.448355 | 7.752453 | 2.25E-06 | 0.000719 | 5.217224 | Up |
| CENPE | 0.603569 | 2.664988 | 7.707272 | 2.40E-06 | 0.000754 | 5.153942 | Up |
| NDC80 | 0.664047 | 2.752792 | 7.601827 | 2.80E-06 | 0.00082 | 5.005123 | Up |
| BIRC5 | 0.603493 | 2.707786 | 7.480018 | 3.36E-06 | 0.000929 | 4.831228 | Up |
| PBK | 0.908888 | 2.830885 | 7.447574 | 3.53E-06 | 0.000934 | 4.78455 | Up |
| CCNA2 | 0.649891 | 2.786368 | 7.431572 | 3.61E-06 | 0.000934 | 4.761472 | Up |
| MAD2L1 | 0.641047 | 2.824503 | 7.270359 | 4.61E-06 | 0.001045 | 4.526888 | Up |
| E2F8 | 0.764043 | 2.515364 | 7.195743 | 5.16E-06 | 0.001137 | 4.417026 | Up |
| FOS | -0.78104 | 3.128878 | -7.18654 | 5.24E-06 | 0.001137 | 4.403417 | Down |
| RAD51AP1 | 0.609137 | 2.637122 | 6.780126 | 9.87E-06 | 0.001647 | 3.789989 | Up |
| FOXM1 | 0.718864 | 2.779688 | 6.658173 | 1.20E-05 | 0.00182 | 3.601093 | Up |
| FOSB | -0.71232 | 2.93264 | -6.1179 | 2.91E-05 | 0.003262 | 2.737201 | Down |
| MCM10 | 0.615531 | 2.553813 | 6.048872 | 3.27E-05 | 0.003623 | 2.623637 | Up |
| RERGL | -0.73729 | 2.651899 | -5.91067 | 4.13E-05 | 0.004189 | 2.394128 | Down |
| CDKN3 | 0.611386 | 2.884551 | 5.765524 | 5.30E-05 | 0.004893 | 2.149993 | Up |
| OSR1 | -0.6391 | 2.565819 | -5.17616 | 0.00015 | 0.009183 | 1.127146 | Down |
| ECRG4 | -0.65189 | 3.060458 | -5.08731 | 0.000176 | 0.009944 | 0.968717 | Down |
| IL33 | -0.68459 | 2.745338 | -4.90295 | 0.000247 | 0.012219 | 0.63669 | Down |
| PTGS2 | -0.62578 | 2.25146 | -4.7074 | 0.000355 | 0.015158 | 0.279867 | Down |
| ID4 | -0.60157 | 2.74923 | -4.62963 | 0.000411 | 0.016381 | 0.136715 | Down |
| NUF2 | 0.754377 | 2.767897 | 4.603767 | 0.000431 | 0.016806 | 0.088947 | Up |
| OGN | -0.69366 | 2.973987 | -4.59442 | 0.000439 | 0.017038 | 0.071664 | Down |
| KERA | -0.59414 | 2.254204 | -4.58842 | 0.000444 | 0.017106 | 0.060575 | Down |
| MMP1 | 0.975554 | 2.637231 | 4.427865 | 0.000601 | 0.02059 | -0.23775 | Up |
| NEK2 | 0.588507 | 2.724049 | 4.268963 | 0.000814 | 0.024896 | -0.53547 | Up |
| PRAME | 0.628158 | 2.648352 | 3.991168 | 0.001391 | 0.033741 | -1.06081 | Up |
| CXCL11 | 0.786017 | 2.817473 | 3.951815 | 0.001501 | 0.034996 | -1.13564 | Up |
| ST8SIA6.AS1 | 0.635623 | 2.648881 | 3.53002 | 0.003427 | 0.055957 | -1.9411 | Up |
| CXCL13 | 0.635373 | 3.04029 | 3.411898 | 0.004324 | 0.062247 | -2.16693 | Up |
| COL11A1 | 0.602355 | 2.995839 | 2.81467 | 0.014 | 0.117345 | -3.29582 | Up |
|  |  |  |  |  |  |  |  |
